# Is a Healthy Diet Also Suitable for the Prevention of Fragility Fractures?

**DOI:** 10.3390/nu12092642

**Published:** 2020-08-30

**Authors:** Eva Warensjö Lemming, Liisa Byberg

**Affiliations:** Department of Surgical Sciences, Section of Orthopedics, Uppsala University, SE- 751 83 Uppsala, Sweden; liisa.byberg@surgsci.uu.se

**Keywords:** fragility fracture, dietary pattern, epidemiology, prevention, diet

## Abstract

Osteoporosis and sarcopenia contribute to the risk of fracture in the population. These conditions share common features, and it is known that a healthy diet may have beneficial effects on both, theoretically resulting in fewer fractures. The present narrative review gives an overview of recent epidemiological research related to the association between healthy diets/dietary patterns, bone health and fragility fractures. The review also gives a brief overview on general dietary recommendations and advice as the cornerstone of public health nutrition. Although muscle health and sarcopenia contribute to the risk of fractures, these endpoints were not the focus of this review. Healthy diets are nutrient dense and contain bioactive components that are needed for the constant remodeling of the skeleton and to slow the rate of bone loss and muscle wasting, thus contributing to the prevention of fragility fractures. Compliance with healthy dietary patterns were predominantly found to be inversely associated with bone outcomes, although this was not entirely consistent across all studies. Different a priori diet scores, such as the Mediterranean diet score and the Dietary Inflammatory Index, as well as a posteriori data driven dietary patterns, such as the prudent or healthy dietary pattern, were inversely associated with fragility fractures in different populations. In conclusion, different healthy dietary patterns may contribute to bone health and less fractures. Following current dietary guidelines is thus advisable for the prevention of fragility fractures.

## 1. Introduction

Age-related bone loss [1] and sarcopenia [2,3] are two important determinants that contribute to the risk of falls and fragility fractures. These conditions share common features and it is known that a healthy diet may have beneficial effects on both osteoporosis and sarcopenia, theoretically resulting in fewer fragility fractures [4]. Hip fracture is the most severe type of fragility fractures and is associated with a high burden of morbidity, healthcare costs and mortality [5]. Because of the burden of fragility fractures both for the individual and society, preventive strategies for primary prevention such as lifestyle modifications, are important to consider. Diet is one modifiable lifestyle factor that may help prevent fragility fractures. A diet comprises of a wide range of foods and individual dietary components have been related to hip fractures; for example, a higher intake of vegetables and fruits was inversely associated [6,7] while a higher consumption of soda was associated with an increased risk [8]. However, people consume foods as part of a diet that may theoretically be collapsed into dietary patterns, which in turn can be used in the study of diet–disease associations. The aim of the present narrative review is to give an overview of recent epidemiological research related to the association between dietary patterns, bone health and fragility fractures. Although muscle health and sarcopenia contribute to the risk of fractures, these endpoints are not the main focus of this review, but will be part of the discussion. The association between dietary patterns, skeletal muscle health, and sarcopenia has recently been reviewed [9]. The present review also aims to give a brief overview on general recommendations and advice regarding healthy diets as the cornerstone of public health nutrition for the prevention of late-onset diseases, with specific emphasis on bone health and fragility fractures. 

## 2. Diet and Dietary Patterns

In a diet, all food substances, nutrients and bioactive compounds, are consumed as foods rather than the individual components, and this food matrix may deliver effects beyond the sum of its individual components [10,11]. Healthy diets comprise different food combinations with common core foods influenced by regional food culture [12,13]. To be able to study diet, reflective of the food matrix, dietary pattern analysis is used. There are mainly two ways to generate dietary patterns from collected diet data in a population, a posteriori data driven methods, such as principal component analysis (PCA) or cluster analysis, or using a priori formed indices or diet scores [14]. For example, PCA produces continuous dietary pattern variables, which are reflective of actual reported intakes and do not necessarily represent the most healthy or unhealthy pattern in the population [15]. Diet scores reflect empirically derived dietary patterns [14] and are often influenced by official dietary recommendations for example the Diet Quality Index and the Healthy Eating Index [16], but also the regional context for example the Mediterranean diet score [17]. Both ways of studying dietary patterns have strengths and weaknesses. Foremost, the generation of dietary patterns attempts to group food and/or nutrient components into collapsed exposure variables to be used in the study of diet–disease associations, which is a strength. When using a priori index scores, careful consideration has to be given to which diet variables to include, which cut-offs to use for the selected variables and how the final index should be handled in the analyses. For a posteriori dietary patterns, the grouping of the dietary data into food groups has to be carefully done, and there are also subjective decisions to be made during the generation of the patterns, although the statistical program forms the dietary patterns. In all studies, the generated dietary index or pattern will be a reflection of diet data, sometimes making comparisons between studies difficult because of differences in the original dietary data as well as the data management [14,17,18].

## 3. Dietary Recommendations, Food Based Dietary Guidelines, Late-Onset and Non-Communicable Chronic Diseases

Suboptimal diet is an important preventable risk factor for chronic diseases. According to a recent study on the global burden of disease, an improved diet could potentially prevent one in every five deaths globally. Suboptimal intake of three dietary factors (low intake of whole grains and fruits, and a high intake of sodium) was estimated to account for more than 50% of deaths and 66% of disability-adjusted life years (DALYs) attributable to diet [19]. Thus, improved dietary quality on a population level could have major implications for public health and contribute to individual wellbeing. A recommended healthy diet according to current recommendations, for example the Nordic Nutrition Recommendations [20] and the dietary guidelines for Americans [21], is characterized by fruits and vegetables, whole grains, nuts, fish, low fat dairy and vegetable fat, as well as a low intake of red and processed meats, refined grains, sugars and saturated fat. Such a diet may reduce the risk of non-communicable chronic diseases, for example, cardiovascular disease and diabetes [14,22]. 

Dietary recommendations are cornerstones of public health nutrition. Dietary recommendations and reference values are published throughout the world and take a number of forms. Dietary reference values are set for apparently healthy populations to meet physiologic needs, minimize risk of adverse effects, and to decrease the risk of chronic diseases at different life stages. The reference values are used for planning and assessing diets in populations and to inform other policy documents and measures, for example food-based dietary guidelines [20,23,24]. 

The scientific base for dietary recommendations rests on the evaluation of the relevant research. Systematic reviews form the basis of contemporary recommendations; they collect, appraise, and synthesize the body of evidence, using predefined transparent methods [24]. These methods include steps to evaluate bias of individual studies, and then to appraise the strength of causality of the association [23]. A key challenge in evaluating the association between food substances and chronic disease outcomes is that there is a lack of randomized controlled trials (RCT), designed to establish causality. Thus, most evidence on the associations between food substances, health outcomes and chronic diseases comes from prospective cohort and other observational studies, with study design limitations making it difficult to determine causality in some instances [23]. However, RCTs are far from being the only solution in the study of food substances/diet and chronic disease, since the results of RCTs may also be biased. A key reason for this is that the exposure to food substances is complex, with interactions and synergies between dietary components. In addition, there is the behavioural exposure of diet, making high compliance and blinding difficult. It may also be difficult to define a valid comparator to a food. RCTs are often short-term and include selected groups of participants, making the generalizability of the results to the general population difficult [25]. Although an RCT may have excellent internal validity, it does not necessarily mean that the results may be useful for setting dietary reference values [26]. As stated by Satija et al. “given the complex nature of the human diet, another way of inferring causality is to consider different types of exposure (i.e., dietary patterns, foods, nutrients, and biomarkers) simultaneously”. Further, in assessing the association between food components and chronic disease, evidence from several types of studies are considered, and causality is strengthened when different types of studies provide consistent evidence [25].

Food-based dietary guidelines translate the evidence base regarding associations between food substances and health outcomes and disease into specific and culturally appropriate dietary guidelines, which incorporate country-specific evidence and other legitimate factors. Examples of other evidence are food and nutrient intakes, food supplies, the prevalence and public health importance of diet related health and nutrition outcomes, as well as the cultural context [27]. Food safety issues may also be incorporated into advice to the population. For example, in Sweden, consuming fish two to three times per week is recommended as part of a healthy diet. However, there are also advice to limit the intake of certain fish in several population groups, because of their content of dioxins and Polychlorinated Biphenyls (PCBs) [28]. Another aspect of the diet that is now considered in food based dietary guidelines are aspects of sustainability [29,30]. Food-based dietary guidelines are primarily intended to influence consumer behaviour to make healthy food choices, and there are consistencies in these across the globe, especially regarding the consumption of plant foods [27]. A recent review describes the concepts and features of a contemporary healthy diet [31].

## 4. Risk and Prevention of Fragility Fractures and Bone Health 

Between 2000 and 2015, both the number and proportion of DALYs attributed to musculoskeletal disorders, osteoporotic fractures being a major contributor, significantly increased [32]. Musculoskeletal disorders was the second most contributing factor of years lived with disability (YLD) in 2015 [32]. This increase is largely due to the secular trend of a growing population with an increasing proportion of older people, a trend that will continue [33]. Osteoporosis is one of the major causes of fragility fractures in individuals over the age of 50 years, and is a silent disease until a fracture occurs. Low bone mineral density (BMD) is a risk factor for fractures and more fractures at older age occur in individuals with a normal or osteopenic BMD, rather than a BMD in the osteoporotic range [34]. From twin studies, it has been estimated that 50–85% of the variation in BMD at middle-age is genetically determined [35]. However, at older ages the heritability of both bone loss [36] and fractures is modest [37]—an indication that lifestyle factors are becoming of greater importance with increasing age. As we grow older there is also an increased tendency for sarcopenia and falls, and thereby a risk of fractures [38]. In the prevention of fragility fractures, diet, physical activity, and other lifestyle exposures such as smoking, alcohol, medicines, and hormones may affect the future risk. Both diet and physical activity are modifiable by intervention and form the basis of public health recommendations for bone health [39]. Although diet is not a cure, it may slow the degeneration of bone and muscle tissue and thereby reduce the risk of falls and fractures. It is also known that both cardiovascular disease [40] and type 2 diabetes [41] may increase the risk of fractures. Thus, prevention of these diseases may also lessen the risk of fractures. However, there are several different pathways as to why a diet high in essential nutrients and other bioactive components may casually contribute to the maintenance of healthy bone and muscle tissue, and thereby help to reduce the risk of fragility fractures.

## 5. Diet and Bone Health

Bone is constantly remodelled through the process of coupled bone turnover. Osteoclasts resorb bone, and osteoblasts fill in the resorptive pits with new bone. Bone tissue is made up by about 35% protein, largely collagen, and 65% mineral, largely as calcium and phosphorus hydroxyapatite crystals. Through the incorporation into bone tissue, or by regulating the resorptive or formative processes in the different bone cells, many nutrients are needed to maintain bone health [42]. The constant remodelling occurs in order both to replace old and damaged bone, and to maintain long term calcium homeostasis. Vitamin D is needed to improve intestinal calcium absorption and renal conservation of calcium to maintain normal concentrations of calcium (and phosphate). If serum calcium is reduced, skeletal calcium will be used as a resource. If calcium is depleted from the skeleton over several years, in a cumulative manner, this may lead to osteoporosis and subsequent risk of fracture [43]. However, there seems to be no extra need of supplemental calcium to avoid fractures if the supply of calcium from the diet is adequate [44,45,46]. Likewise, supplementation with vitamin D for fracture prevention is not needed for healthy community-dwelling adults [44]. Bone loss is not only a result of impaired calcium homeostasis. Other pathogenic mechanisms that contribute to age-related bone loss are inflammation and sequential or concomitant oxidative stress [1]. Oxidative stress acts on bones by several mechanisms including the promotion of bone cell apoptosis. Moreover, osteoblast and osteoclast recruitment and activity can be affected by different cytokines. The inflammatory cytokines, for example, tumor necrosis factor α and the interleukins 1, 6 and 17, promote the generation of osteoclasts and their activity, while at the same time inhibit osteoblast differentiation and function [47]. These processes may also contribute to the loss of muscle mass and the development of sarcopenia [2,3].

**Figure 1:**
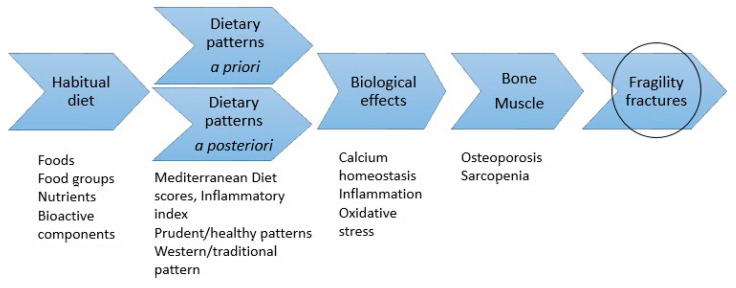
Schematic representation of the theme of the current review; association between diet and fragility fractures. Information on habitual diet is used to create dietary patterns. The dietary patterns are theoretical reflections of the food matrix. The food matrix contains nutrients and other bioactive components necessary for the maintenance of bone and muscle strength and function, thus influencing fragility fracture risk. In light of the literature, the absence of a healthy diet appears to be related to an increased risk of fragility fractures.

It is suggested that both bone and muscle mass progressively decrease by 1–2% per year after the age of 50 years [2]. Thus, to maintain bone health, that is, to support the constant remodeling of the skeleton, as well as to slow the rate of bone loss and muscle wasting due to inflammation and oxidative stress, a continuous influx of different nutrients is needed. Besides calcium and vitamin D, protein, magnesium, and potassium, as well as other micronutrients and trace elements including boron, selenium, iron, zinc, and copper are involved in maintaining healthy bones [42]. A higher vitamin C intake has been linked to higher BMD at the femoral neck and lumbar spine, and a lower fracture risk. Vitamin C is needed for collagen synthesis and for the generation of osteoblasts. Interestingly, vitamin C is also a marker of fruit and vegetable intake and healthy dietary patterns, as well as being an antioxidant [48], which all contribute to the observed beneficial associations with bone health. Other micronutrients with antioxidant properties include vitamins A and E, copper, zinc, and selenium [31]. A high intake of vitamin A, high levels of serum retinol [49,50] and a low intake of alpha-tocopherol, as well as low serum concentrations of alpha-tocopherol [51,52], have been found to predict fracture risk. Moreover, there are mechanisms, reviewed elsewhere [53], by which the different types of dietary fatty acids may influence bone health. Systematic reviews and meta-analyses have provided evidence that saturated fatty acids are positively [54], while n-3 fatty acids [55] are inversely, related to hip fracture risk. Another component of the diet that may influence fracture risk is sugar [56], which possibly has detrimental effects on bone. However, a diet high in sugars is often part of a nutrient poor energy dense dietary pattern, which is associated with a higher risk of non-communicable chronic diseases [22] and has been reported to be associated with a lower BMD [57]. Figure 1 illustrates a schematic representation of the association between diet and fragility fractures.

## 6. Dietary Patterns and Bone Health

So what is the epidemiological evidence for the association between dietary patterns and bone health and fragility fractures? As a starting point for the current review, the evidence gathered in a scoping review from 2017 [58] was used. This review reported on the association of both data-driven a posteriori and pre-defined a priori (dietary score or indices) dietary patterns, and bone health. The study authors found 49 studies conducted in more than 20 countries, published between 2002 and June 2016, relevant to include in their review. The majority of studies (*n* = 32) had used a data-driven approach to define dietary patterns. Most of the studies were however cross-sectional in design. The bone health outcomes considered were BMD, bone biomarkers, osteoporosis or fracture incidence. The study concluded that findings from studies using dietary patterns not only complement the findings from studies using single nutrients and foods, but could also be more useful for translating the research findings into recommendations and advice. Healthy dietary patterns, emphasizing a frequent consumption of health promoting foods (fruit, vegetables, whole grains, poultry and fish, nuts and legumes, and low-fat dairy products) and de-emphasizing the frequent consumption of unhealthy foods (soft drinks, fried foods, meat and processed products, sweets and desserts, and refined grains), were beneficially associated with bone health. Examples of a priori indices that were found to be inversely associated with the risk of fracture include different Mediterranean Diet Scores and the Alternate Healthy Eating Index. However, the Healthy Eating Index-2010 and Dietary Approaches to Stop Hypertension score were not associated with fracture [58]. However, an individual study published in 2018 using data from two US cohorts; Nurses’ Health Study and Health Professionals Follow-Up Study, reported an inverse association between the Alternate Healthy Eating Index-2010 and Dietary Approaches to Stop Hypertension score and hip fracture in women, but not in men. The association was stronger in women above 75 years [59]. There is clearly a lack of RCTs to establish causality in this area. Thus, it is possible that the beneficial associations between different healthy dietary patterns, fragility fractures and bone health in these observational studies are driven by other healthy characteristics among study participants, for example non-smoking, more physical activity and higher income. These factors or confounders should be controlled for in an observational study [60] and different studies have the opportunity to control for different sets of confounding factors. This may indeed affect the results in a study. However, as pointed out earlier, when assessing the association between food components and disease, evidence from several types of studies are considered, and causality is strengthened when different types of studies provide consistent evidence [25]. 

To find relevant studies published after June 2016 (thus not included in the scoping review), a PubMed search was performed on 3 March 2020, using the following search string [(dietary-pattern* or food-pattern*) AND fracture* AND adults AND cohort] without any year restriction. The search rendered 28 publications, out of which 9 were published after the publication of the scoping review and were relevant for this review. These studies could be divided into three categories based on the type of dietary pattern that was used in the studies. The three categories were two a priori diet scores, Mediterranean diet scores and the Dietary Inflammatory Index, and a posteriori data driven dietary patterns. Two of the retrieved studies were systematic reviews and meta-analyses. The first of these studies appraised the association between the Mediterranean dietary pattern and musculoskeletal health in children, adolescents, and adults [61]. The second review concentrated on a posteriori dietary patterns, BMD and risk of fractures [62]. Study characteristics and results from the seven original studies are tabulated in Table 1. A discussion about these studies divided by respective dietary pattern category follows below. In Table 2, a brief description of the components of the dietary patterns retrieved in this narrative review is presented. Available diet data influence how different dietary patterns are generated, which may indeed affect comparability between studies. The Mediterranean dietary pattern is one pattern that exists in several forms to fit food culture, for example the modified Mediterranean diet score [63]. The table depicts the traditional components that were presented in the publication by Trichopoulou et al. [64]. After submission of our narrative review, another review on nutrients and dietary patterns related to osteoporosis has also been published [65].

### 6.1. A Priori Diet Scores

#### 6.1.1. Mediterranean Diet 

One of the most studied diet scores in association with non-communicable chronic diseases is the Mediterranean diet score [75], and bone health is no exception. The systematic review from 2017 included only three prospective cohort studies, two studies on fracture incidence and one study on sarcopenia incidence. These three studies compared higher vs. lower adherence to a strict criteria of traditional Mediterranean diet scores on disease incidence [61]. This study searched for RCTs and prospective cohort studies published until April 2016. In addition to the systematic review, the study included an evidence map on studies, reporting on the association between a Mediterranean diet and musculoskeletal outcomes, which did not meet the eligibility criteria of the systematic review. For example, studies that used other designs or definitions of the Mediterranean diet than the traditional, were not included in the systematic review. Thus, 15 additional studies were included in the evidence map. Among these, eleven studies reported on bone related outcomes. The overall conclusion from the systematic review [61] was that there was a paucity of research to understand the relationship between the Mediterranean diet and musculoskeletal health. One of the difficulties highlighted is the lack of a common definition of the Mediterranean diet score. This conclusion is in contrast to the finding of the scoping review [58] that reported inverse associations between healthy diet scores, including a Mediterranean type of diet and bone health. Since the publication of the systematic review and evidence map [61], other large studies have proposed inverse relationships between Mediterranean type diets and hip fracture rates. The study by Benetou and coworkers from 2018 [66] included a total of 140,775 adults (82.5 % women) from five cohorts from Europe, including the large study (>70,000 men and women) conducted by our study group [63], and the USA. The study participants were followed-up for a total of 1,896,219 person-years and experienced 5454 hip fractures. There was an inverse association between the Mediterranean diet score and hip fracture, Hazard Ratio (HR) 0.94 (95% Confidence Interval (CI) 0.87–1.01), when comparing the high vs. low quintile. This point estimate is however lower compared to the point estimate in the study conducted by our study group [63]. In this study, participants in the highest compared to the lowest category of the modified Mediterranean diet score had a 22 percent lower hip fracture rate (HR 0.78; 95% CI 0.69–0.89) [63]. On the other hand, two healthy dietary pattern scores reflective of the Mediterranean and the Baltic Sea diets were not significantly associated with BMD in a cross-sectional analysis, although the dietary patterns appeared to capture overall healthy characteristics of a diet. This was a study from Finland that included 65 to 71-year-old women, participating in the interventional prospective Kuopio Osteoporosis Risk Factor and Fracture Prevention study [67]. One explanation for differences in results between studies may depend on the scoring of the Mediterranean diet [76], as well as the exposure range of the score components. Two recent studies (not picked up in the search) support an inverse association between the Mediterranean diet and fracture, but once again they highlight how the context of the study population and the dietary data available influence the scoring of dietary patterns and the association with disease. The first study conducted in the Boston Puerto Rican Health Study showed that in post-menopausal women, associations between a higher Mediterranean diet score and BMD were less pronounced compared to using the Dietary Approaches to Stop Hypertension score. The study also reported a lower likelihood of osteoporosis with a higher Mediterranean diet score, as well as the Dietary Approaches to Stop Hypertension score and the Alternative Health Eating Index [77]. Moreover, in the second recent study conducted in a large European cohort study (~25,000 participants) [78], the Mediterranean diet was inversely associated with both total and hip fracture. In the high compared to low quintile, total and hip fracture incidence was reduced by up to 23% and 21%, respectively. In this study both an alternative and a more traditional Mediterranean diet score were generated. The associations were in fact stronger with the alternative Mediterranean diet score. One of the key differences between the scores was the differential scoring of dairy intake, resulting in a lower calcium intake when scoring high with the traditional Mediterranean diet score [78]. 

#### 6.1.2. Dietary Inflammatory Index

The dietary inflammatory index (DII) has been developed to characterize the inflammatory potential of the entire diet [73] and the association between DII and non-communicable chronic diseases has recently been reviewed [79]. Two studies from the US investigated the dietary inflammatory index (DII^®^) and risk of fractures [68,69], and one of the studies also reported on the association between DII and BMD [69]. The DII considers 45 different food components, including not only the content of micronutrients and macronutrients, but also of other bioactive components of the diet such as flavonoids, spices and tea [80]. Veronese et al. reported that women, but not men, in the highest DII score quintile (more inflammatory diet) compared to the lowest, had a significantly higher risk for fractures (HR 1.46; 95% CI 1.02–2.11). During the eight years of follow-up, 560 individuals experienced a fracture. Fractures were self-reported in the study [68]. The second study that investigated the DII with risk of fracture (defined as hip, lower-arm, and total fracture) and BMD used longitudinal data from the Women’s Health Initiative study [69]. Self-reported hip fractures were confirmed by medical records and centrally adjudicated. The study found that postmenopausal women with a less inflammatory DII score, compared to those with a more inflammatory diet, had lower baseline hip BMD, but lost less BMD at the hip over 6 years. A more inflammatory diet was also associated with increased risk of hip fracture in white women younger than the median age of 63 years (HR, 1.48; 95% CI, 1.09–2.01) [69]. A similar association between the DII and hip fracture was reported in a case-control study from China. The study enrolled 1050 case-control pairs, and the odds ratio of hip fracture, in the highest compared to the lowest quartile of DII, was OR 2.44; 95% CI 1.73–3.45 [81]. 

### 6.2. A Posteriori Dietary Patterns

The systematic review that reported on a posteriori dietary patterns and bone outcomes included studies published until May 2018. The review provided evidence of a favorable influence of dietary patterns referred to as “Prudent/Healthy” on BMD, which was detected in all age groups. There was also evidence of a positive association between dietary patterns referred to as “Western/Unhealthy” and low BMD, but only in older individuals. The meta-analyses indicated an inverse association between “Prudent/Healthy” dietary patterns and risk of fracture among men (Odds Ratio (OR) 0.81; 95% CI 0.69–0.95), while a positive association between “Western/Unhealthy” dietary patterns and fracture (OR 1.10; 95% CI 1.02–1.19) [62]. Other studies that were picked up in the present review further support an inverse association between “Prudent/Healthy” dietary patterns and bone health, while a positive association of a “Western/Unhealthy” pattern. One of the studies was our study investigating a posteriori dietary patterns and registry ascertained hip fractures in women. In this study, we found that women (*n* = 56,736) with a higher adherence to the healthy dietary pattern had a 31% lower hip fracture rate (highest vs. lowest quartile, multivariable adjusted HR 0.69; 95% CI 0.64–0.75). In contrast, women following a more Western/convenience pattern, had a 50% higher rate of hip fracture in the highest versus the lowest quartile (multivariable adjusted HR 1.50; 95% CI 1.38–1.62) [70]. Another study examined an a posteriori dietary pattern and self-reported fractures using data from the China Health and Nutrition Survey. This study reported that a higher adherence to a modern dietary and/or an animal-sourced nutrient pattern was related to a higher risk of total fractures. The modern type of dietary pattern was characterized by high intake of fruits, milk, cake, fast foods, eggs, soy milk and deep fried products, and the animal-sourced nutrient patterns were high in protein, fat, vitamins A, B2 and E, and low in potassium, calcium, magnesium and vitamin C [71]. Thus, these patterns share characteristics with the unhealthy dietary patterns observed in Western countries. Based on data from a multicenter cohort based in the US, Rogers et al. reported an inverse association between adherence to a prudent dietary pattern and BMD change at the total hip, but not at the femoral neck, over 4 years. The Western dietary pattern was not significantly associated with a consistent BMD change at either site [72]. 

## 7. Conclusions

The current narrative review suggests that consuming a varied healthy diet is associated with beneficial effects on bone health, and could be important for the prevention of fragility fractures. There is however not only one way of eating healthy. The review found three major categories of dietary patterns, published after 2016, that were related to risk of fractures and BMD. The three categories were two a priori diet scores; Mediterranean diet scores and the Dietary Inflammatory Index, and a posteriori data driven dietary patterns. A healthy compliance with each dietary pattern was predominantly beneficially associated with bone outcomes, although this was not entirely consistent across all studies. Thus, a healthy diet may be constructed in different ways, which depends on the available diet data in the population at study. Despite this, results are quite consistent. A reason for this may be that different dietary patterns reflect common aspects of a healthy diet. As an example, the Mediterranean diet score used in the publication of Byberg et al. [63] and the a posteriori derived healthy dietary pattern 1997 reported by Warensjö Lemming et al. [70] correlated quite well (r = 0.62, unpublished results). A higher adherence to both these healthy dietary patterns were related to a lower hip fracture rate. Core characteristics of a healthy diet are higher intake of vegetables and fruits and other plant foods; however, there is inconclusive evidence that a stricter vegetarian diet may be deleterious for bone health [82], which should be further explored.

Inconsistencies in results between studies may however be due to methodological aspects such as study size, the number of fractures, fracture ascertainment, the exposure width, and losses to follow up. Healthy diets are nutrient dense and also contain bioactive components needed to stay healthy, for the constant remodeling of the skeleton and to slow the rate of bone loss and muscle wasting due to inflammation and oxidative stress. Following current dietary guidelines is thus also advisable for the prevention of fragility fractures.

## Figures and Tables

**Table 1:** Depicted is the study characteristics of the seven studies published after April 2016 with relevance for the present narrative review.

Study, Location, and Design (Reference)	Participant Information	Follow-Up Time Outcome	Results
Mediterranean dietary patterns			
Benetou et al. [66] Cohorts in Europe and USA, Chances project	Men and women *n* = 140,775 60 years and older.	1,896,219 person-years 5454 incident hip fractures	Comparing high with low adherence; Pooled HR = 0.96; 95% CI, 0.92–0.99.
Erkkilä et al. [67] Finland	Women *n* = 554 Mean age = 67.9 years.	Bone mineral density (BMD) at different sites Cross-sectional analyses	Femoral, lumbar or total body BMD were not significantly different between the quartiles of Baltic Sea Diet or Mediterranean diet score.
Dietary Inflammatory Index			
Veronese et al. [68] USA	Men and women with/at risk of knee osteoarthritis. *n* = 3648 Mean age = 60.6 years	8 years of follow-up 560 self-reported fractures at hip, spine, and forearm.	Comparing highest quintile with the lowest; In women, HR = 1.46; 95% CI, 1.02–2.11. In men, HR = 0.91; 95% CI, 0.54–1.54. In all participants, HR = 1.22; 95% CI, 0.91–1.64.
Orchard et al. [69] USA	Postmenopausal Women *n* = 160,191 Mean age = 63 years.	11.3 years of follow-up. Bone mineral density (BMD) and 47,974 self-reported incident fractures. Including 3837 centrally adjudicated hip fractures.	Those defined with a less inflammatory DII score had lower hip BMD at baseline, but lost less BMD at the hip over 6 years. Comparing the highest quartile with the lowest; In women, younger than 63 years and white: HR, 1.48; 95% CI, 1.09–2.01, Similar non-significant trend for older women.
Warensjö Lemming et al. [70]. Sweden	Women *n* = 56,736 Median age = 52 years.	Median follow-up time of 25.5 years. 4997 registry ascertained hip fractures.	Comparing the highest with the lowest quartile; Healthy pattern, HR = 0.69 95% CI, 0.64–0.75. Western/convenience pattern, HR = 1.50; 1.38–1.62.
Melaku et al. [71] China	Men and women *n* = 15,572 18 years or older	Median follow-up time of 8.9 years. 649 self-reported incident fractures.	Comparing the high with the low tertile. Cumulative scores of the modern dietary pattern, HR = 1.34, 95% CI: 1.06–1.71 and the animal-sourced nutrient patterns, HR = 1.37, 95% CI: 1.08–1.72.
Rogers et al. [72] USA	Men *n* = 4379 Mean age 72.9 years.	Longitudinal change over 4 years in hip bone mineral density (BMD)	Higher adherence to the Prudent dietary pattern was modestly associated with decreased BMD loss at the total hip in older men. The Western dietary pattern was not associated with BMD change.

HR: Hazard ratio, CI: confidence interval. All HRs reported in the table were multivariable adjusted.

**Table 2:** Brief description of the dietary patterns discussed in the current review.

Type of Dietary Pattern	Dietary Pattern	Components	References
*A priori dietary patterns Pre-defined items which are scored depending on intakes.*	Mediterranean dietary pattern	Olive oil, vegetables, fruit, cereals, legumes, nuts, fish, dairy products, egg. potatoes	[64]
	Dietary inflammatory index	Nutrients, tea, antioxidants, pices	[73]
*A posteriori dietary patterns Generated in a data-driven approach*	Healthy or prudent dietary pattern	Fruit, vegetables, fish, poultry, low-fat dairy products, cereals, nuts, seeds	[74]
	Western or traditional dietary patterns	Processed foods, red meat, sausages, butter, French fries, eggs, high-fat dairy products, soda	[74]
